# Cumulative contact frequency of a chromatin region is an intrinsic property linked to its function

**DOI:** 10.7717/peerj.9566

**Published:** 2020-08-10

**Authors:** Margarita D. Samborskaia, Aleksandra Galitsyna, Ilya Pletenev, Anna Trofimova, Andrey A. Mironov, Mikhail S. Gelfand, Ekaterina E. Khrameeva

**Affiliations:** 1 Faculty of Bioengineering and Bioinformatics, Lomonosov Moscow State University, Moscow, Russia; 2 Center of Life Sciences, Skolkovo Institute of Science and Technology, Moscow, Russia; 3 A.A. Kharkevich Institute for Information Transmission Problems, RAS, Moscow, Russia; 4 Institute of Gene Biology, RAS, Moscow, Russia

**Keywords:** Hi-C, Chromatin, Compartments, Conformation capture

## Abstract

Regulation of gene transcription is a complex process controlled by many factors, including the conformation of chromatin in the nucleus. Insights into chromatin conformation on both local and global scales can be provided by the Hi-C (high-throughput chromosomes conformation capture) method. One of the drawbacks of Hi-C analysis and interpretation is the presence of systematic biases, such as different accessibility to enzymes, amplification, and mappability of DNA regions, which all result in different visibility of the regions. Iterative correction (IC) is one of the most popular techniques developed for the elimination of these systematic biases. IC is based on the assumption that all chromatin regions have an equal number of observed contacts in Hi-C. In other words, the IC procedure is equalizing the experimental visibility approximated by the cumulative contact frequency (CCF) for all genomic regions. However, the differences in experimental visibility might be explained by biological factors such as chromatin openness, which is characteristic of distinct chromatin states. Here we show that CCF is positively correlated with active transcription. It is associated with compartment organization, since compartment A demonstrates higher CCF and gene expression levels than compartment B. Notably, this observation holds for a wide range of species, including human, mouse, and *Drosophila*. Moreover, we track the CCF state for syntenic blocks between human and mouse and conclude that active state assessed by CCF is an intrinsic property of the DNA region, which is independent of local genomic and epigenomic context. Our findings establish a missing link between Hi-C normalization procedures removing CCF from the data and poorly investigated and possibly relevant biological factors contributing to CCF.

## Introduction

The conformation of chromatin in the nucleus plays an important role in many cellular processes, including the regulation of gene transcription and DNA replication (Cremer et al., 2006; Sexton et al., 2007; Bickmore, 2013; Armstrong et al., 2018; Fulco et al., 2019). The regulation of gene expression often involves long-range chromatin interactions between regulatory elements. Therefore, the spatial organization of chromatin could provide insight into these complex regulatory processes.

Hi-C is a method for genome-wide chromosome conformation capture, which enables the interrogation of all loci at once by combining DNA proximity ligation with high-throughput sequencing (Lieberman-Aiden & Van Berkum, 2009). However, data obtained by Hi-C have both technical and experiment-induced biases. Because of that, different regions of the genome may have different visibility in the experiment, yielding systematic errors in data interpretation. To correct for this bias, several approaches exist, reviewed in Lajoie, Dekker & Kaplan (2014) and Schmitt, Hu and Ren (2016). Some recent advances in the Hi-C data analysis allow for various modifications of the correction procedure, such as probabilistic modeling (Yaffe & Tanay, 2011), Vanilla-Coverage (Lieberman-Aiden & Van Berkum, 2009), binless normalization (Spill et al., 2019) and other. One of the most commonly used methods for the elimination of systematic biases is the iterative correction (IC) (Imakaev et al., 2012). In particular, it is used as a gold standard in the Hi-C data processing package *cooler* (Abdennur & Mirny, 2019).

While IC is based on the assumption that all loci have equal visibility (observed number of contacts), the differences in experimental visibility may be explained not only by technical or experimental biases but also by biological factors. Notably, local chromatin conformation correlates with functional characteristics of the genome, such as individual histone modifications (Khrameeva et al., 2012) or their combinatorial patterns that establish certain functionality for each region, chromatin states (Ernst et al., 2011). Therefore, elimination of the differences in the visibility of chromatin regions could lead to the loss of a biologically meaningful signal.

Features such as TADs, enriched contacts, and compartments are usually called in normalized Hi-C interaction maps (Forcato et al., 2017). Experimental visibility is treated as a purely methodological artifact that is assumed not to affect the detection of these features. Other studies (Chandradoss, Guthikonda & Kethavath, 2020; Beagrie et al., 2017), have previously highlighted the importance of experimental visibility of DNA regions in Hi-C. However, to our knowledge, the relation of this genomic characteristic to expression and chromatin states has not been analyzed. Here, we establish a relation between visibility of DNA in Hi-C, assessed by the cumulative contact frequency (CCF), and chromatin states in a range of species.

## Methods

### Analysis of Hi-C data

#### Processing of Hi-C data

We analyzed Hi-C maps for human cell lines HMEC, HUVEC, and K562, mouse cell line CH12-LX (Rao et al., 2014), and fruit fly (Ulianov et al., 2015) Schneider-2 (S2) cells (GEO database, accession numbers GSE63525 and GSE69013, respectively). We downloaded the processed Hi-C maps in the hic format from (Rao et al., 2014) and in the txt format from (Ulianov et al., 2015). The Hi-C maps were converted to the matrix format and binned at the 1 Mb resolution. The main results were obtained for the human cell line HMEC and, where possible, for other cell lines and species to demonstrate the generalizability of our findings (see Supplemental Information).

To eliminate possible technical artifacts of Hi-C, such as single-sided reads and their subsets, mirror reads (Galitsyna et al., 2017), even though the coverage profile for these reads might be well-correlated with the coverage profile of double-sided reads (Imakaev et al., 2012), we removed the diagonal 1-Mb elements of the Hi-C maps. Additionally, for CCF calculations, we removed the secondary diagonal corresponding to regions immediately adjacent to each other and all contacts at the distance up to 5 Mb in order to remove the area of high contact frequencies that could hinder subsequent analysis. Genomic regions corresponding to rows and columns of Hi-C maps, which contained no values, were also removed from all analyses.

We calculated the cumulative contact frequency (CCF) as the sum of contact frequencies of each locus. To make CCF comparable between different cell lines and resolutions, we further report it as the percentage from the maximum CCF in the Hi-C map. We considered two types of CCF: whole-genome and inter-chromosomal (calculated for inter-chromosomal Hi-C maps). Inter-chromosomal CCF was analyzed separately to demonstrate that intra-chromosomal contacts do not drive our observations.

#### TAD and compartment calling

We used the *Armatus* algorithm (Filippova et al., 2014), as implemented in the *Lavaburst* package (accessed 01-12-18, *modularity* scoring function and *gamma* parameter 1.0 (Abdennur, 2018)), for TAD calling in human Hi-C maps at the 1 Mb resolution. We considered all segments smaller than three bins as interTAD regions. This allowed us to classify the genomic bins into two categories: TAD and interTAD bins. We then used these bins separately for the correlation analysis of CCF at TAD and interTAD genomic regions.

In order to identify chromatin compartments, we performed computational analysis as in Lieberman-Aiden & Van Berkum (2009). For that, we normalized the whole-genome contact matrix by the expected contact frequency matrix, generated by averaging contact probabilities for loci at each genomic distance. We then calculated the Pearson correlation coefficients for each row/column pair of each element of the normalized matrix to obtain the correlation matrix. The resulting correlation matrix was then used for the principal component analysis (PCA). We used the first principal component of the resulting correlation matrix as a compartment annotation for the genome.

Notably, the first principal component for human and mouse datasets demonstrated the highest proportion of variance explained (PVE of the first component ranging from 0.60 for HMEC and HUVEC cells to 0.80 for K562 cells) and had a characteristic checkerboard pattern in accordance with previous findings (Lieberman-Aiden & Van Berkum, 2009; Rao et al., 2014). We were unable to detect compartments in the analyzed *Drosophila* dataset (PVE for the first component of S2 cells is 0.11), probably due to *Drosophila* compartments being much smaller than the selected dataset resolution (1 Mb).

### Functional characteristics

We estimated the functional characteristics of genomic regions by combinatorial patterns of chromatin marks, or chromatin states, for human (Ernst et al., 2011), mouse (Yue et al., 2014), and *Drosophila* (Kharchenko et al., 2011). These chromatin states were originally derived from a set of ChIP-seq experiments for various chromatin factors by Hidden Markov Models, and represented distinct states with specific ChIP-seq signatures. Chromatin states are better for the assessment of functional properties of genomic regions than individual marks from two perspectives. First, they represent an integrated view of the region’s expression and functional characteristics; the experimental noise of individual ChIP-seq experiments is smoothed out. Second, the analysis of chromatin states is simpler, compared to a set of marks.

We retrieved fifteen states from Ernst et al. (2011) for the human genome, seven states for the mouse genome (Yue et al., 2014), and nine states for the *Drosophila* genome (Kharchenko et al., 2011). The original datasets were downloaded in the format of a non-intersecting set of genomic regions, with a unique chromatin state assigned to each region. In order to match the Hi-C data uniform grid, we segmented the genome into non-overlapping 1-Mb genomic windows, or bins, starting from the first position of each chromosome. For each genomic bin, we then computed the fraction of coverage of each chromatin state. If the initial chromatin state segment spanned the bin boundary, it was split into two parts by the bin boundary and counted as contributing to both bins that it overlaps, proportionally to the resulting fragments sizes. Thus, for each bin and chromatin state, we obtained a single number from 0 to 1, reflecting the coverage of this bin by the chromatin state. Bins containing no annotation of chromatin states were removed from further analysis.

The chromatin states for the human genome from Ernst et al. (2011) are named by the principal function of the respective regions. We separated them into two groups by functional activity. The first group is active chromatin: Active Promoter, Weak Promoter, Inactive/poised Promoter, Strong Enhancer (2), Weak/poised Enhancer (2), Weak Transcription, Transcriptional Elongation, Transcriptional Transition. The second group is inactive chromatin: Repetitive/CNV (2), Heterochromatin.

Chromatin states for mouse from Yue et al. (2014) are named by the histone modifications prevalent in the corresponding state. The active marks are represented by: H3K4me3, H3K4me1/3, H3K4me1, H3K4me1+H3K36me3, and H3K36me3. Only one state, H3K27me3, represents inactive chromatin, and one state is comprised of all unmarked genomic regions.

The states for the *Drosophila* genome are called ”colors” with functional load described in the original publication (Kharchenko et al., 2011). Based on that, we separated *Drosophila* states into two groups, active chromatin, comprized of RED (1) and MAGENTA (2) colors, and inactive/repressed chromatin, comprized of DARKGRAY (6), DARKBLUE (7), LIGHTBLUE (8), LIGHTGRAY (9).

### Correlation analysis

To characterize correlation patterns, we used two approaches. First, we calculated the Pearson correlation coefficients between CCF and chromatin state proportions in each region of the whole genome. To further validate the findings, we used Stereogene (Stavrovskaya et al., 2017), a tool for the genome-wide feature correlation analysis. We explored the relationship between pairs of characteristics of the genome, such as CCF, GC-content, and proportion of each chromatin state. Stereogene divides the input data into a series of fixed-length windows (adjustable parameter that was set to 10 Mb), and the independent correlation is calculated for each set. The distribution of these correlations allows one to observe the variation in the correlation coefficient across the genome and to identify regions with non-typically high positive or negative correlation. These distributions are compared against a randomized control derived from the data (Stavrovskaya et al., 2017), and *p*-values are calculated for the observed correlations in the real data.

### Analysis of syntenic regions

Syntenic regions (size 2 Mb and larger) were obtained from the Mouse Genome Informatics database (MGI) (Finger et al., 2011). The regions of homology with the human genome (size 1 Mb) were established using the *LiftOver* tool (Hinrichs, 2006). The contact frequency for ambiguously mapped regions was split proportionally to the lengths of the mapped fragments. For this analysis, we defined large chromosomes as chromosomes 1–9, and small chromosomes as chromosomes 14–22. The Pearson correlation coefficient was calculated between the human and mouse CCF.

## Results

### Increased CCF is associated with active transcription

**Figure 1:**
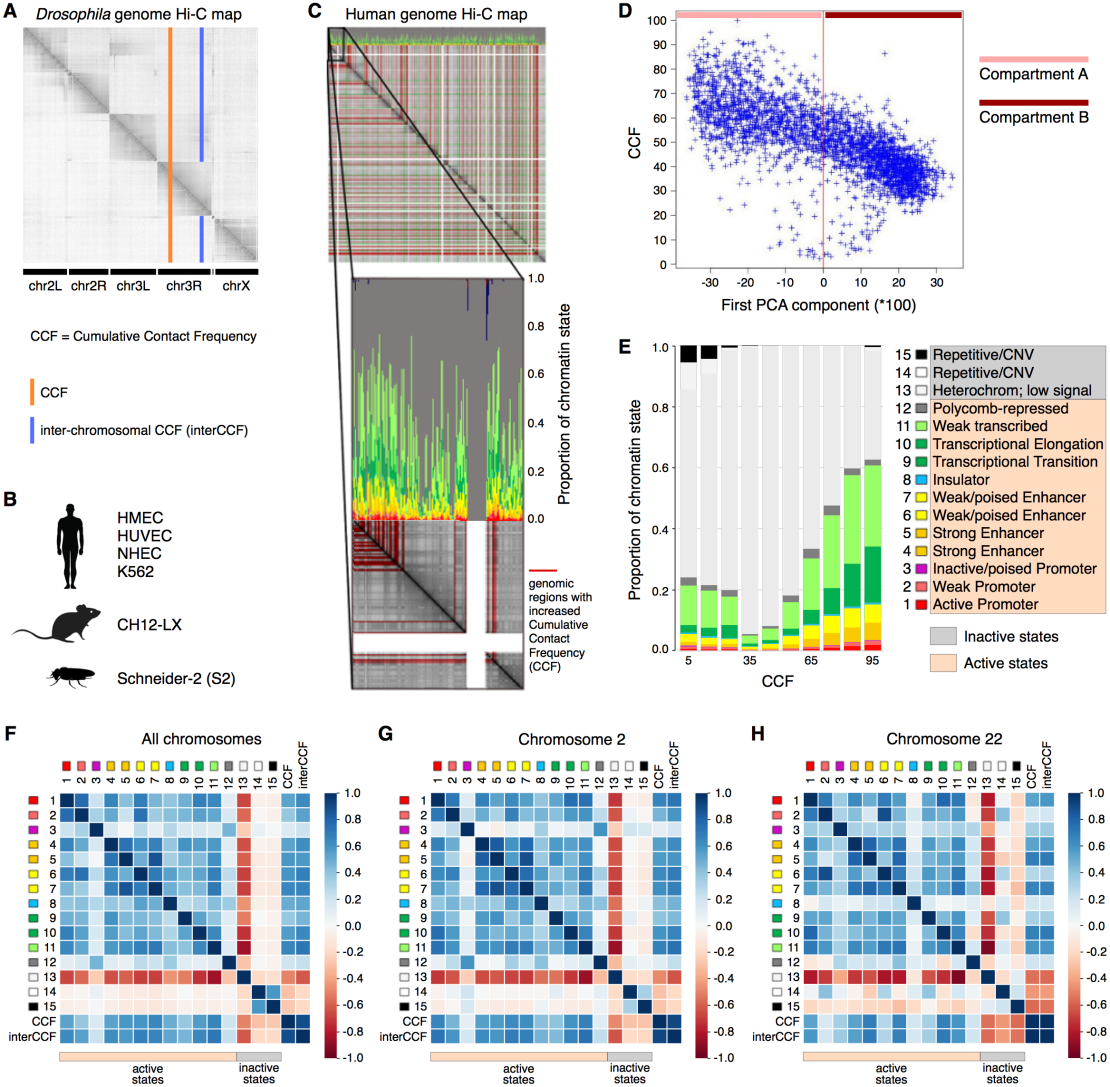
Cumulative contact frequency (CCF) is positively correlated with active transcription. (A) Schematic representation of inter-chromosomal (blue line) and total (orange line) CCF. (B) Cell lines and organisms analyzed in this study. (C) Hi-C map combined with a plot of chromatin state proportions. Red lines on the Hi-C map show regions of anomalously high CCF. Green lines separate individual chromosomes. Proportions of each chromatin state for each genomic region are displayed above the Hi-C map. An enlarged fragment of the Hi-C map for chromosome 1 is shown below. (D) Dependency of CCF on the first principal component. (E) Dependency of chromatin state proportions on CCF. (F-H) Correlation patterns between chromatin states and CCF exhibit different features for large and small chromosomes. First 15 rows in the matrix correspond to the 15 chromatin states, rows 16-17 exhibit total and inter-chromosomal CCF. Colors demonstrate the Pearson correlation coefficients. Whole-genome correlation patterns (F), correlation patterns for chromosome 2 (G) and chromosome 22 (H) are shown. Human cell line HMEC.

As active transcription requires binding of RNA polymerase and a variety of transcription factors, increased gene expression is intuitively associated with loose packaging of chromatin and thus better accessibility to the Hi-C reaction and higher CCF. At the same time, active chromatin is involved in a larger number of interactions, including distant regulatory ones. Thus, one might expect regions with high CCF to show high gene expression levels and regions with low CCF to exhibit low gene expression. To validate this hypothesis, we constructed a whole-genome Hi-C map combined with the functional state plot showing the distributions of chromatin state proportions for each genomic region (Figs. 1A–1C). Indeed, for all analyzed human cell lines (HMEC, HUVEC, and K562), the regions with high CCF tend to be enriched in chromatin states corresponding to active transcription, while regions showing low CCF are enriched in heterochromatin and repeats (Fig. 1C, Fig. S1).

Chromosomes are known to segregate into two mutually exclusive types of chromatin, referred to as “A” and “B” compartments (Lieberman-Aiden & Van Berkum, 2009). Active chromatin corresponds to the A compartment, while repressed chromatin is enriched within the B compartment. Using correlation analysis of normalized Hi-C maps and PCA, we segregated the genome into two types of chromosomal regions. We observe that human compartment A has high levels of CCF in HMEC and other human cell lines (Fig. 1D, Fig. S2).

### CCF is linked to active chromatin states

To get a more precise estimate of dependencies between CCF and chromatin states, we visualized the growth of chromatin state percentages at increasing CCF (Fig. 1E, HMEC cells). We observe the growth of percentages of the chromatin states corresponding to active transcription (Weak Transcription, Transcriptional Elongation, and Transcriptional Transition, in particular) with larger CCF. This result does not depend on the Hi-C data resolution, as proved by the same analysis repeated for 1 Mb, 500 Kb, 250 Kb, 100 Kb, and 50 Kb resolutions (Fig. S3).

To further validate the result, we calculated the correlations between each of the chromatin states and CCF (Fig. 1F) enabling comparative analysis of different genomic regions. CCF is positively correlated with active chromatin state proportions in HMEC cells (correlation coefficient 0.53). The same result is obtained for other human cell lines (Fig. S1): HUVEC (correlation coefficient 0.47) and K562 (correlation coefficient 0.35). As an additional proof of concept, the homogeneity of correlations across the genome was confirmed for the cell line HMEC (Fig. S4) with the Stereogene tool (Stavrovskaya et al., 2017). Notably, the correlation patterns are similar for large chromosomes but different for smaller ones (Fig. 1F, Fig. S5).

To show that the dependencies between CCF and chromatin states are not specific to humans, we additionally analyzed the *Drosophila* cell line S2 and mouse cell line CH12-LX. For *Drosophila* and mouse, the chromatin state annotations (Kharchenko et al., 2011) differ from that in human. In particular, there are fewer chromatin states, and their functional characteristics are different. However, for *Drosophila*, we observe a positive correlation of CCF with chromatin states RED (1) and MAGENTA (2) (Fig. S6), which are representative of active chromatin with expressed genes. For mouse, we observe a positive correlation of CCF with all chromatin states but the one characterized by absence of chromatin marks (Fig. S7).

TADs and interTAD regions demonstrate different patterns for the human genome (Fig. S8). TAD CCF is correlated with active chromatin and anti-correlated with inactive chromatin, while interTAD CCF is correlated with heterochromatin and insulator chromatin states. The latter fact might be related to the interTAD insulator property. By contrast to humans, TADs and interTAD regions have only slight differences in *Drosophila* (Fig. S9), where both TAD and interTAD CCF demonstrate a positive correlation with active chromatin states and a negative correlation with inactive chromatin states.

### CCF association with active chromatin is not driven by GC-content

The observed correlation between CCF and chromatin states is not necessarily direct and causative, as there might exist other genetic or epigenetic factors underlying both CCF and active chromatin state. If there is such a confounding factor, then accounting for its influence would diminish the observed correlations.

One possible type of confounders are GC-content and chromosome length. Our initial analysis demonstrates that GC-content and chromosome length are indeed both correlated with contact frequency, and the dependencies are linear or nearly linear (Fig. S10–Fig. S11). Inter-chromosomal CCF decreases with chromosome length, which indicates that small chromosomes tend to make more inter-chromosomal contacts than large chromosomes, in line with previous studies showing that small chromosomes are gene-rich and tend to interact with each other (Fig. S11A) (Lieberman-Aiden & Van Berkum, 2009). In particular, the correlation between the chromosome length and an average inter-chromosomal CCF is -0.42 for the cell line HMEC.

To test whether CCF is correlated with active chromatin state in the absence of these confounding factors, we performed a simple division of CCF by these factors and re-calculated the correlation plots. CCF normalized by the chromosome length or by the GC-content demonstrated the same correlation patterns as non-normalized CCF (Figs. S10B, S11B). To further validate this observation, we applied linear regression to predict CCF from the GC-content. The correlation patterns are weakened, but still the same as for non-normalized CCF (Fig. S10B). Further, normalization of CCF by the chromosome length and subsequent removal of the GC-content effect shows that, even combined, the chromosome length and GC-content cannot explain the observed correlation patterns (Figs. S10B, S11B).

### CCF for different chromosomes reveals hidden variability in correlation patterns

Each chromosome has its own unique properties, which cannot be detected while considering the correlation pattern for the whole genome. Since each chromosome differs in contact frequency preferences, the correlation patterns calculated for separate chromosomes may also differ. Indeed, while the first nine chromosomes show a correlation pattern similar to that of the whole genome, smaller chromosomes exhibit individual unique correlation patterns (Figs. 1G–1H, Fig. S5).

**Figure 2:**
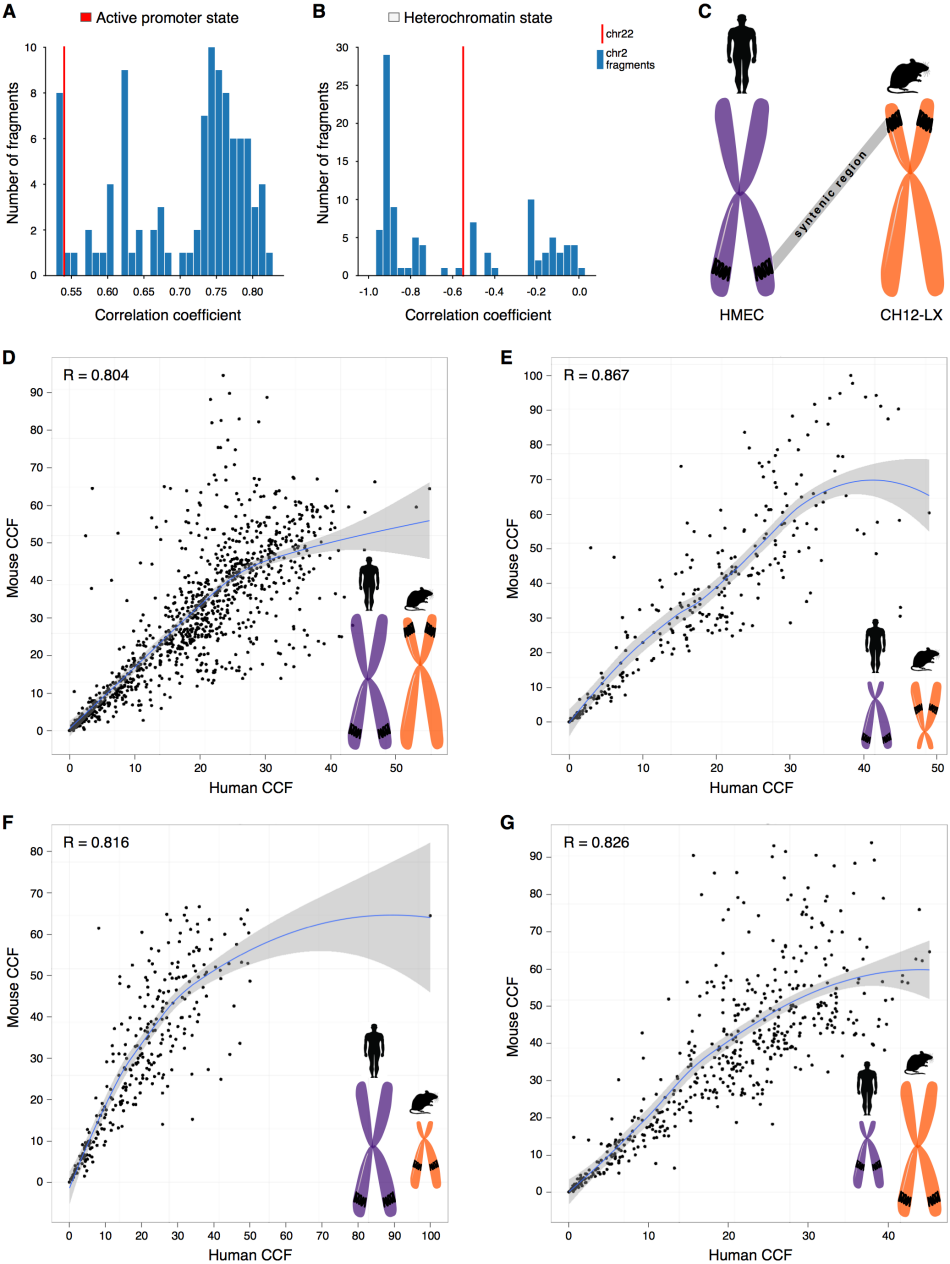
CCF is a position-independent inherent property of chromatin regions conserved in syntenic transitions. (A–B) Distribution of correlations between CCF and active promoter state (A) or heterochromatin state (B) for random fragments of chromosome 2 (blue bars) is compared with the real correlation for chromosome 22 (red line). Human cell line HMEC. (C) Schematic representation of a syntenic region between two chromosomes of the human and mouse genomes. Human cell line HMEC and mouse cell line CH12-LX. All syntenic regions of size 1 Mb are obtained by mapping the mouse genome to the human genome using the Liftover tool. (D–G) CCF in human versus CCF in mouse for syntenic regions in large human chromosomes and large mouse chromosomes (D), small human chromosomes and small mouse chromosomes (E), large human chromosomes and small mouse chromosomes (F), small human chromosomes and large mouse chromosomes (G). Each dot represents a syntenic region (size 1 Mb).

One possibility is that we have observed a statistical artifact, caused simply by differences in the sample size, as, naturally, more fragments are considered for large chromosomes than for small ones. However, downsampling large chromosomes to the size of small chromosomes demonstrates that correlations of small chromosomes still are outliers (Figs. 2A–2B, Fig. S12). It suggests that the observed effects for small chromosomes are not due to the small sample size.

We have noticed that another important factor might be the size of centromeres, which might have different sizes hence forming different fractions of chromosomes. We have excluded the centromere regions and demonstrated that the observed correlation patterns are not related to differences in the centromere size (Fig. S13).

Notably, for individual chromosomes of the *Drosophila* genome, the correlation patterns are more similar (Fig. S6). However, a direct comparison with the results for human is impossible due to differences in chromatin state annotations between the human and *Drosophila* datasets.

### Comparison of CCF in syntenic blocks between mouse and human

Small chromosomes might show unique correlation patterns due to the impact of specific evolutionary conserved regions, such as syntenic blocks. To test this hypothesis, we annotated syntenic regions and calculated CCF for them in the human and mouse datasets.

Indeed, syntenic regions of short chromosomes demonstrate correlations between contact frequency and chromatin states that are not characteristic of syntenic blocks in long chromosomes (Figs. 2A–2B, note the difference between real correlations (red line) and controls (blue bars)). Moreover, syntenic regions have similar preferences in contact frequencies between two species (Figs. 2C–2E).

To understand how syntenic regions inherit the properties during genomic rearrangements in evolution, we identified syntenic blocks located in small chromosomes of the human genome, but in large chromosomes of the mouse genome, and visa versa. Notably, these regions exhibit similar CCF in the human and mouse genomes (Figs. 2F–2G, the correlation between the contact frequencies in the human and mouse genomes ranges from 0.82 to 0.83). Thus, the observed correlation preferences are intrinsic properties of syntenic blocks as they do not depend on the location of the region in the genome and are inherited despite evolutionary rearrangements between chromosomes (Fig. 2, Fig. S14–Fig. S15).

## Discussion

Data normalization is a typical step of Hi-C data processing that corrects hidden biases of the interaction signal (Lyu, Liu & Wu, 2019; Calandrelli et al., 2018; Sauria et al., 2015). One of the most widely used normalization methods is the iterative correction (IC), which assumes equal visibility of each genomic region in the experiment. Various features of Hi-C maps, such as TADs, enriched contacts and compartments, are called after the step of normalization. However, the equal visibility assumption might result in removal of biologically relevant information obtained from Hi-C. We sought to dissociate the technical and biological signal that is removed by IC.

Here, we introduce cumulative contact frequency (CCF) for a genomic region as the number of contacts for a region in a non-normalized Hi-C map. We then analyze CCF properties, including correlation with biologically meaningful signals such as chromatin compartments, transcriptional activity, and chromatin states.

We observe that for human cells, large CCF is predictive of active chromatin and compartment A. This result holds for multiple resolutions of the Hi-C data and several human cell types. We also have used the Stereogene approach (Stavrovskaya et al., 2017) to demonstrate that the correlations are reproduced for the subsets of genomic regions.

Moreover, positive correlation of CCF with active chromatin states holds for *Drosophila* and mouse, suggesting broad generalizability of our conclusions. Notably, we use human and mouse Hi-C that were mapped by Rao et al. (2014) and *Drosophila* Hi-C that was mapped by Ulianov et al. (2015) with different data processing pipelines. We find it striking that the general correlations of CCF are independent of the details of the upstream data processing, which is supportive of the biological importance of CCF. Parallel analysis of CCF properties in multiple cell types demonstrates robustness of the observed correlations, suggests a general similarity between cell types, and further supports the proposed relevance of the CCF signal.

To further separate the biologically relevant signal of CCF from possible technical artifacts, we have considered confounding factors that might affect our analysis. GC-content is a well-known source of variability in the genomic coverage for sequencing experiments, Hi-C, in particular (Yaffe & Tanay, 2011). We have demonstrated that CCF is predictive of active chromatin even after the removal of this confounding factor.

One of the first observations obtained using Hi-C method was the tendency of small chromosomes to interact with each other while being more active (Lieberman-Aiden & Van Berkum, 2009). Thus, CCF might be different for chromosomes of different sizes. In order to control for that, we have used CCF normalized by the chromosome size and demonstrated reproducibility of the observed correlation patterns.

Surprisingly, we have observed that CCF of small and large chromosomes differs. We suggest that this difference might happen not because of the chromosome size, but because of the intrinsic properties of the regions. First, we have confirmed it by downsampling large chromosomes to the size of small ones. Second, we have compared CCF in syntenic regions between the human and mouse genomes and observed that CCF does not change after translocation between large and small chromosomes.

There are still some other possible technical confounding factors that might contribute to the CCF properties, such as the density of restriction fragments in a genomic bin, mappability of the region, chromatin openness as assessed by DNase-seq or ATAC-seq (Yaffe & Tanay, 2011). These factors remain out of scope of the present research.

Importantly, all these observations do not allow us to introduce a causative link between chromatin activity and CCF. We also do not account for the evolutionary history and sequence conservation of corresponding regions, which might reveal the reasons for our cross-species observations. Extensive further research is required to shed the light on these problems.

Nevertheless, our results allow to suggest that removal of CCF in the IC procedure is currently understudied. CCF contains biologically relevant information that is not affected by GC-content and chromosome size. Currently, the effect of removal of this information on calling of Hi-C features, such as TADs, enriched contacts, and compartments, has not been studied. We propose to take the Hi-C normalization step with caution and interpret Hi-C features that are robust to the removal of CCF and present in both non-normalized and normalized maps.

## Conclusions

In this work, we dissociate the technical and biological signal that is removed by the iterative correction (IC), one of the most widely used methods of Hi-C data normalization. For that, we study cumulative contact frequency (CCF) defined as the number of contacts for a genomic region in a non-normalized Hi-C map. We demonstrate that CCF has significant biological properties, such as correlation with chromatin compartments, transcriptional activity, and active chromatin states. These properties are independent of GC-content and chromosome sizes. They can be generalized to a broad range of species (human, mouse, and *Drosophila*). Surprisingly, these properties are inherited and preserved between syntenic regions of human and mouse genomes. We conclude that the importance of CCF is underestimated, and it should be removed from Hi-C maps with caution.

##  Supplemental Information

10.7717/peerj.9566/supp-1 Supplemental Information 1 Figures S1-S15

## Additional Information and Declarations

### Data Availability

The following information was supplied regarding data availability: We used publicly available data from GEO: GSE63525 and GSE69013.
