# Supplementary material for: Cumulative contact frequency of a chromatin region is an intrinsic property linked to its function
Source: PeerJ. 2020 Aug 10;8:e9566. doi: 10.7717/peerj.9566 (PMC7425636; doi:10.7717/peerj.9566)
Supplement: Supplemental Information 1 [file peerj-08-9566-s001.docx]

**Supplementary Materials**


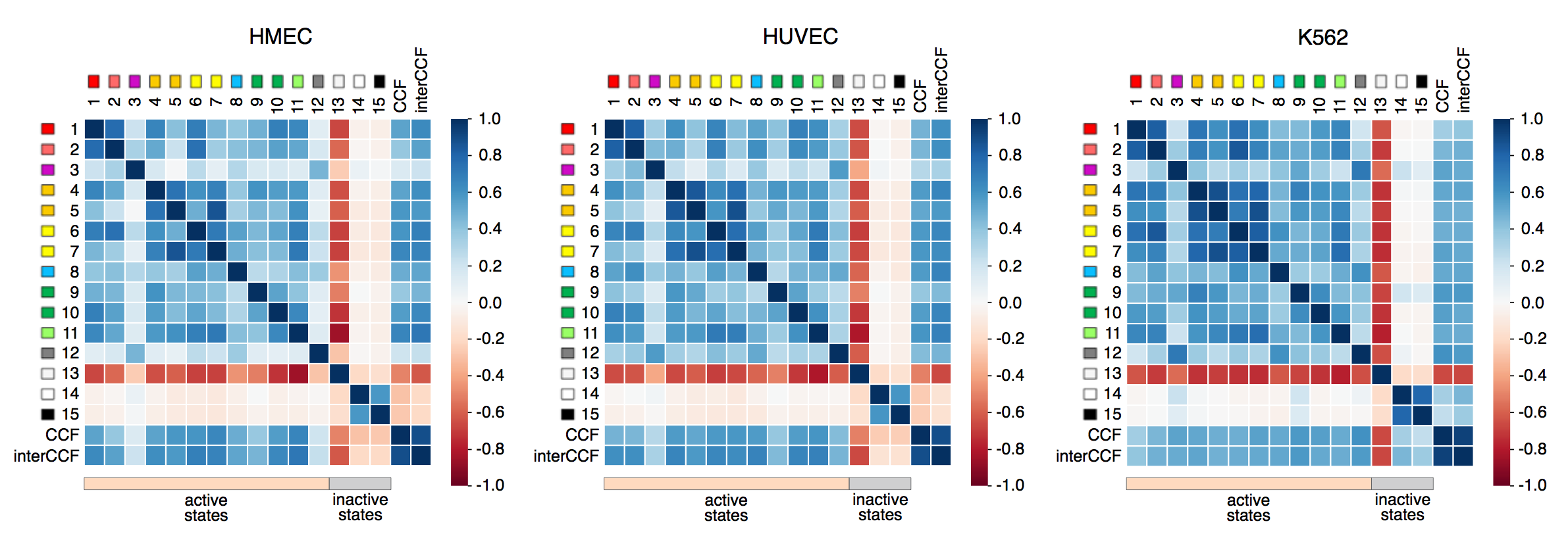


**Supplemental Figure S1. Whole-genome correlation patterns for three human cell lines.** Chromatin states for the human cell lines are correlated with the total CCF and inter-chromosomal CCF. First 15 rows in the matrix correspond to the 15 chromatin states, rows 16-17 exhibit whole-genome and inter-chromosomal CCF. Colors show the Pearson correlation coefficients. Note that correlation patterns are similar for whole-genome and inter-chromosomal CCF.


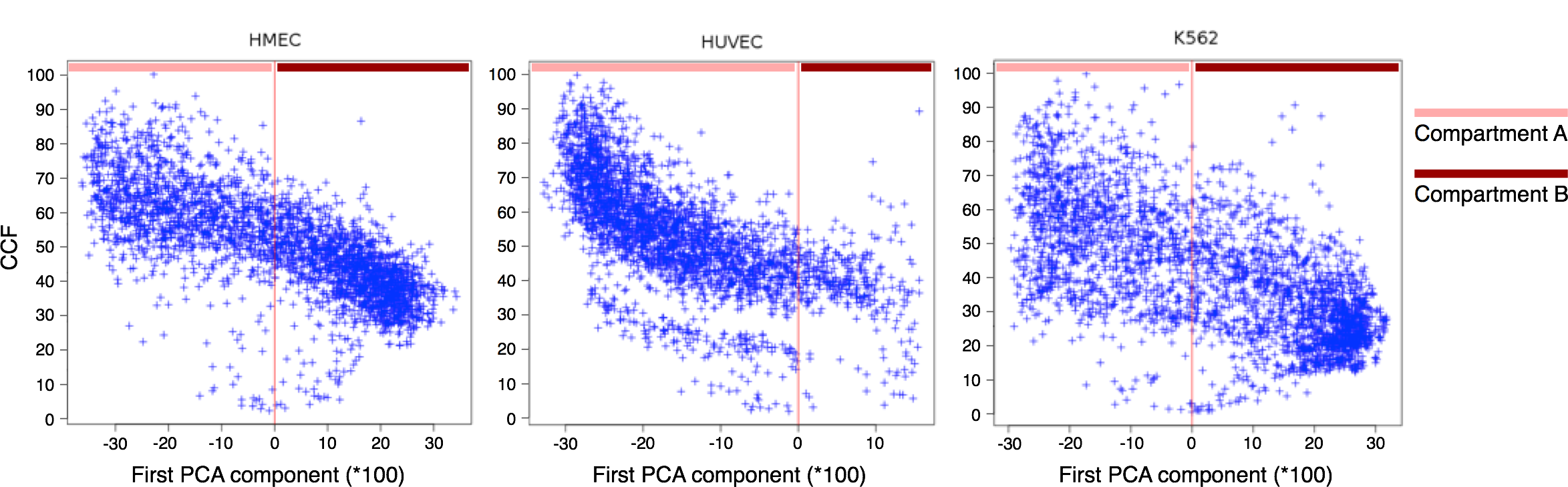


**Supplemental Figure S2. Dependency of CCF on the first principal component.** The first principal component was calculated using PCA analysis of the Hi-C maps at 1 Mb resolution in three human cell lines: HMEC, HUVEC, and K562.

**
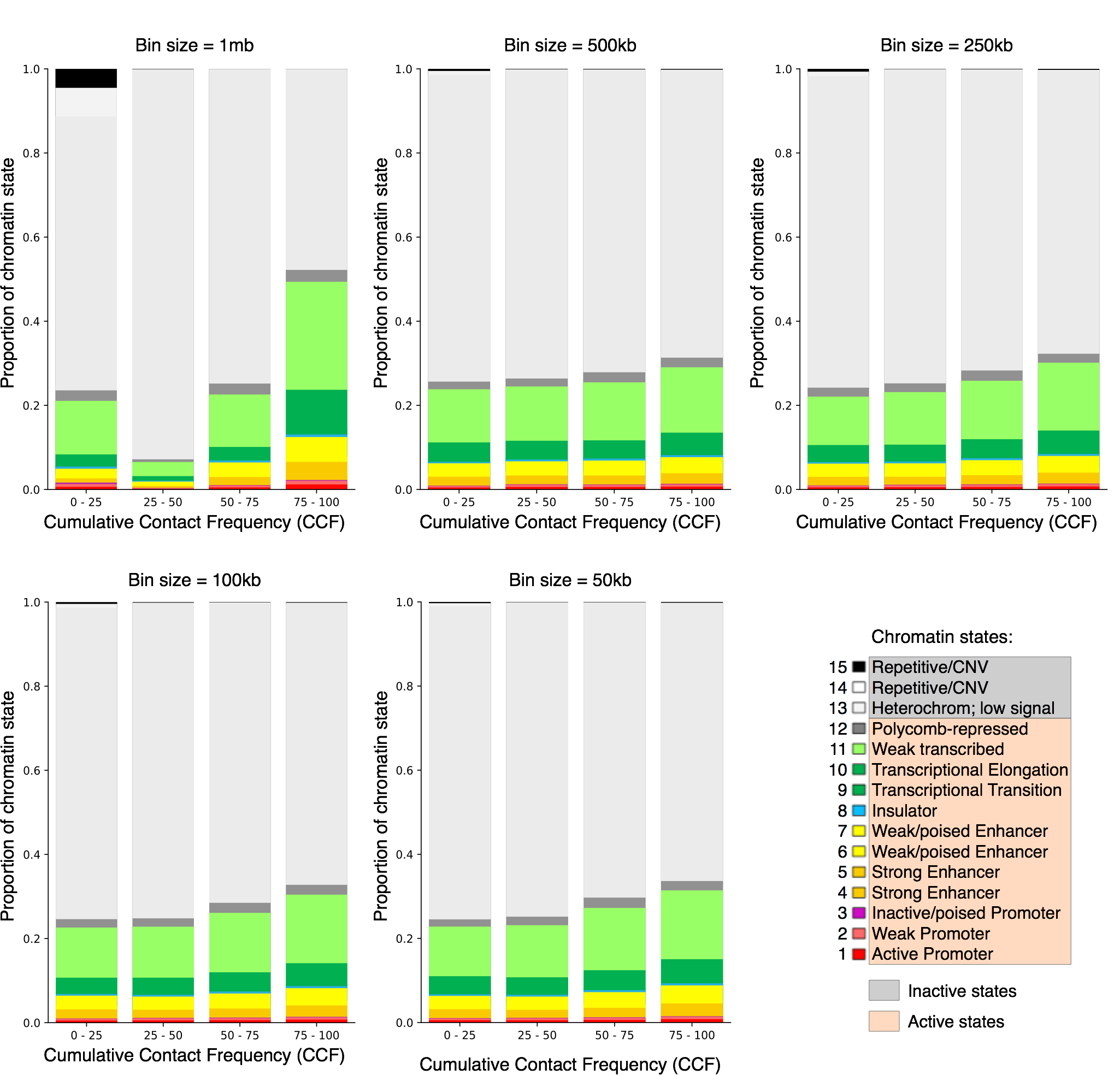
**

**Supplemental Figure S3. Dependency of chromatin state proportions on CCF.** CCF and chromatin states were calculated for five resolutions (bin sizes) – 1 Mb, 500 Kb, 250 Kb, 100 Kb and 50 Kb. Human cell line HMEC.


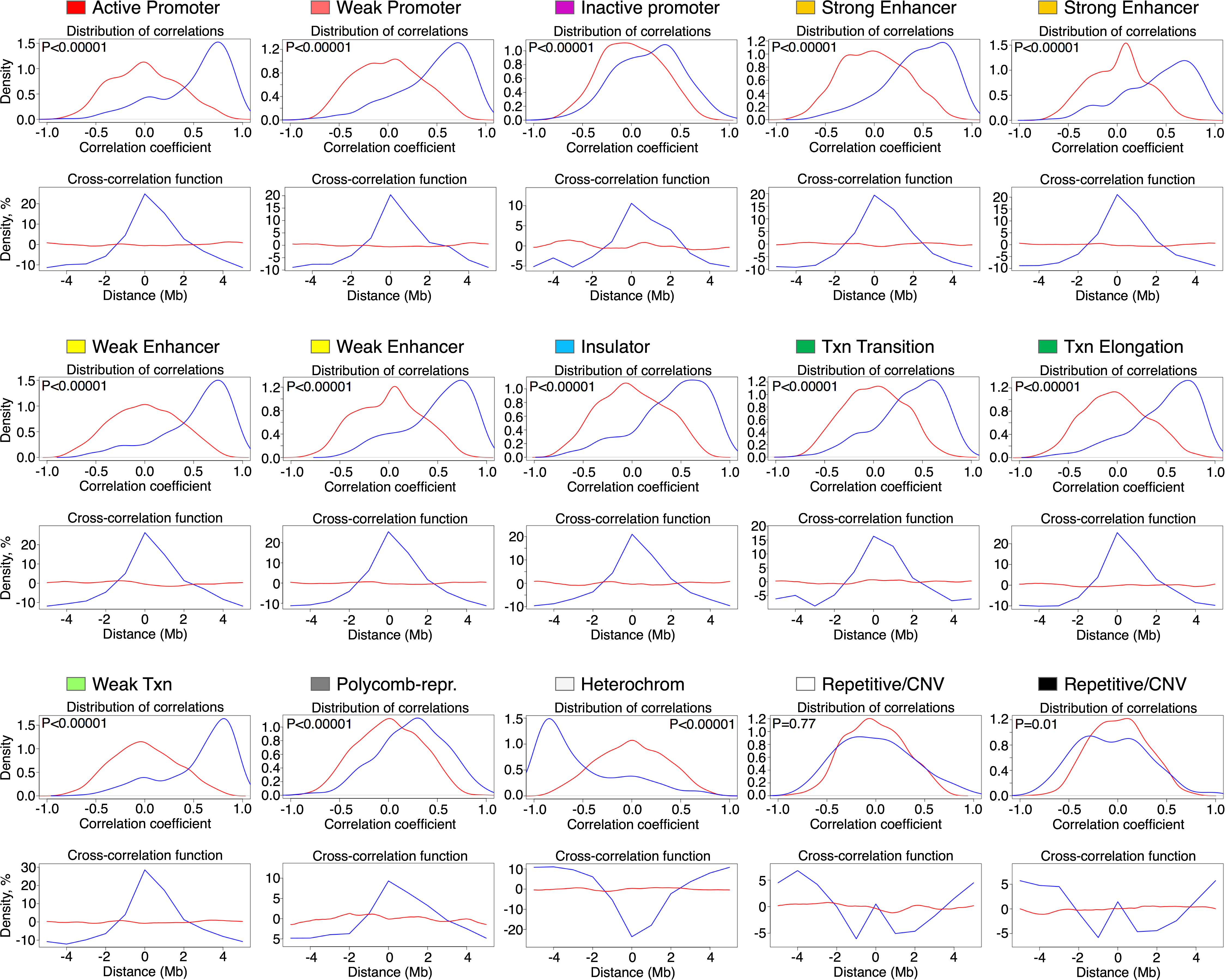


**Supplemental Figure S4. Validation of correlations between chromatin states 1 to 15 and whole-genome CCF with the Stereogene tool.** The distribution of real correlations (blue line) is compared with randomly selected windows (red line). Stereogene parameters except for the window size (size 10 Mb) were set to default. For each chromatin state, the top panel shows the distribution of correlations, while the bottom panel shows the cross-correlation function. Human cell line HMEC.


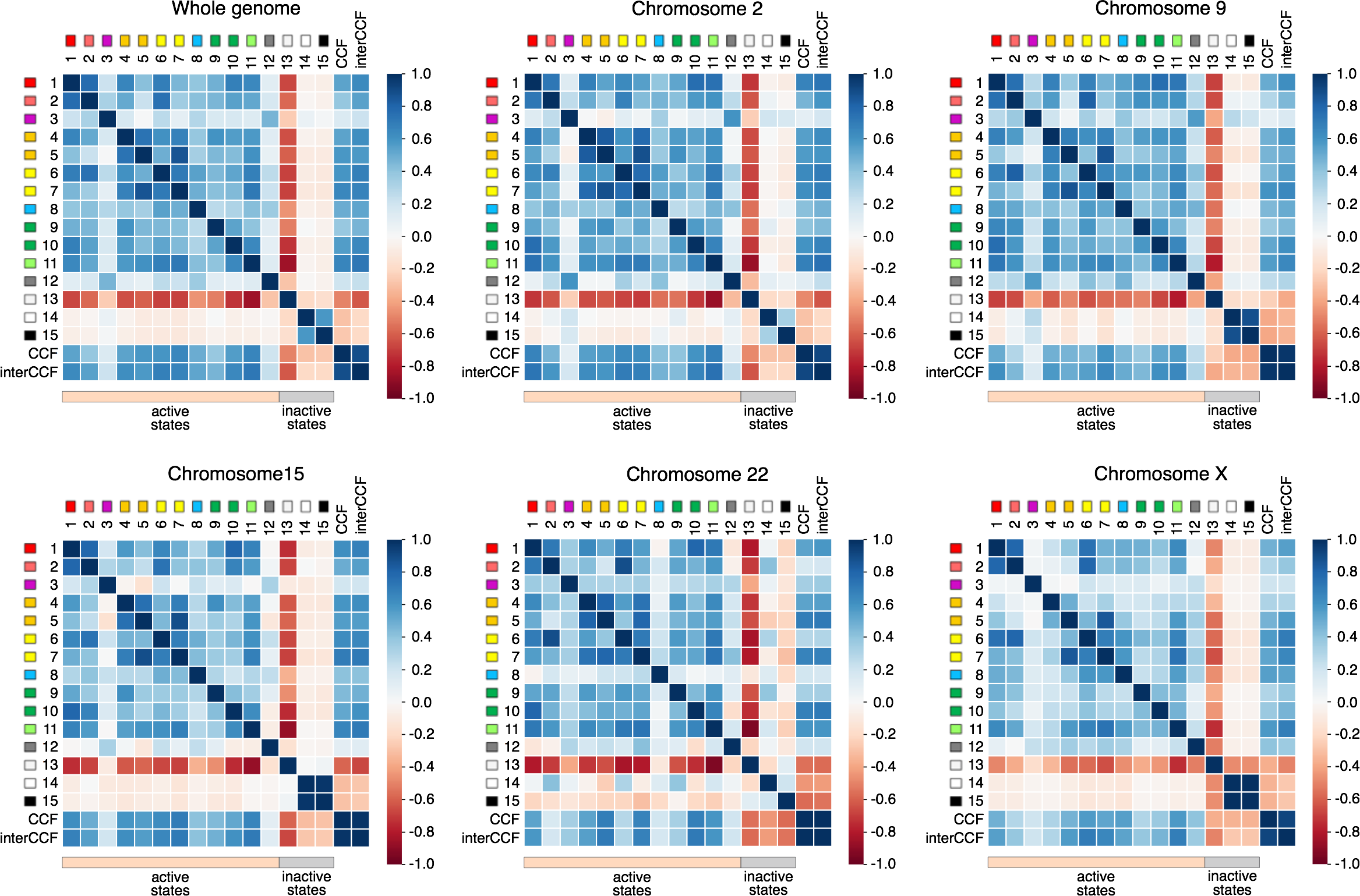


**Supplemental Figure S5. Correlation patterns for different human chromosomes.** Note that correlation patterns are similar for large chromosomes, but are different for small chromosomes. Human cell line HMEC.


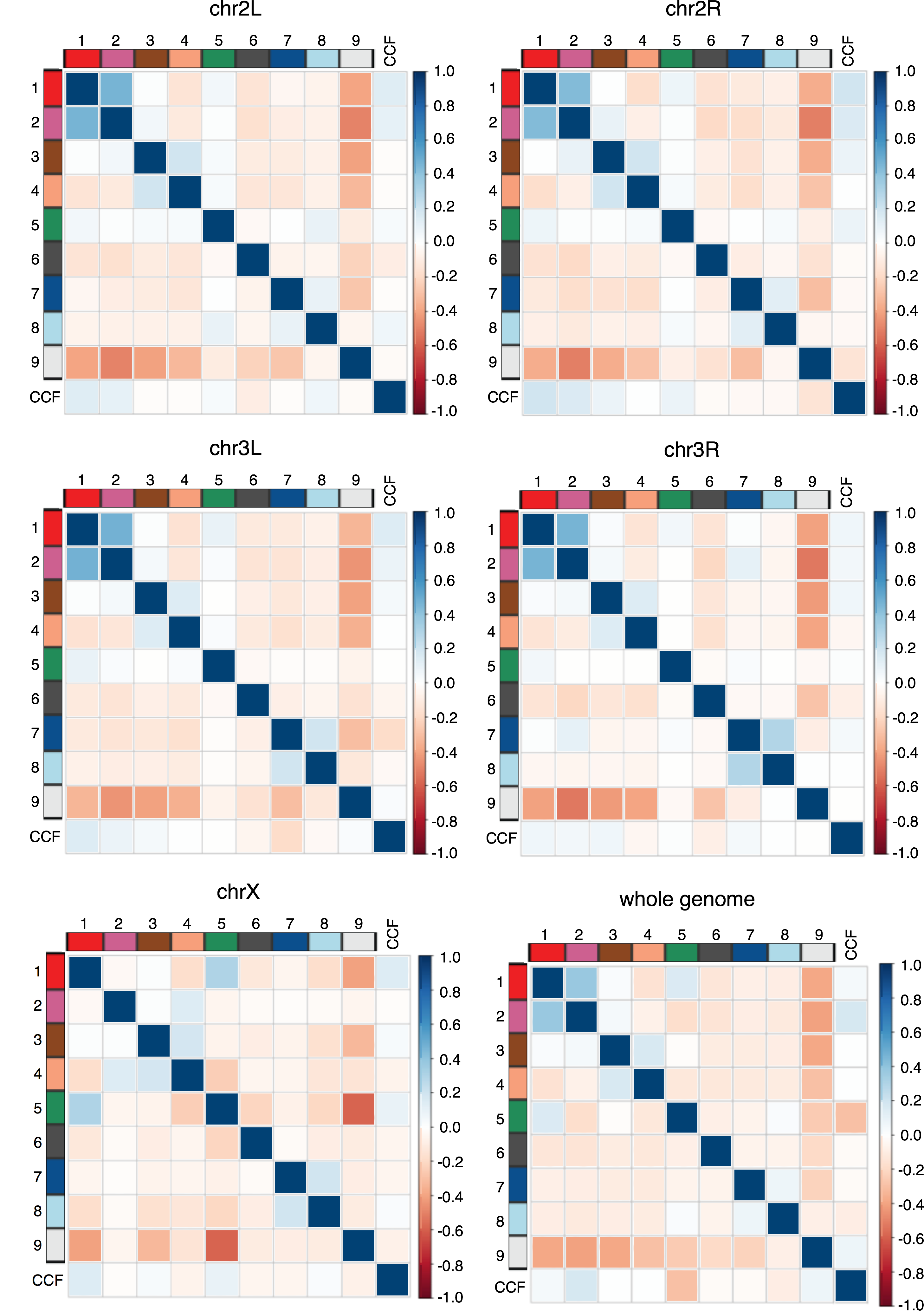


**Supplemental Figure S6. Correlation patterns of whole-genome CCF and chromatin states in *Drosophila*.** The correlation patterns are similar for the whole genome and individual chromosomes. *Drosophila* cell line S2-DGRC.


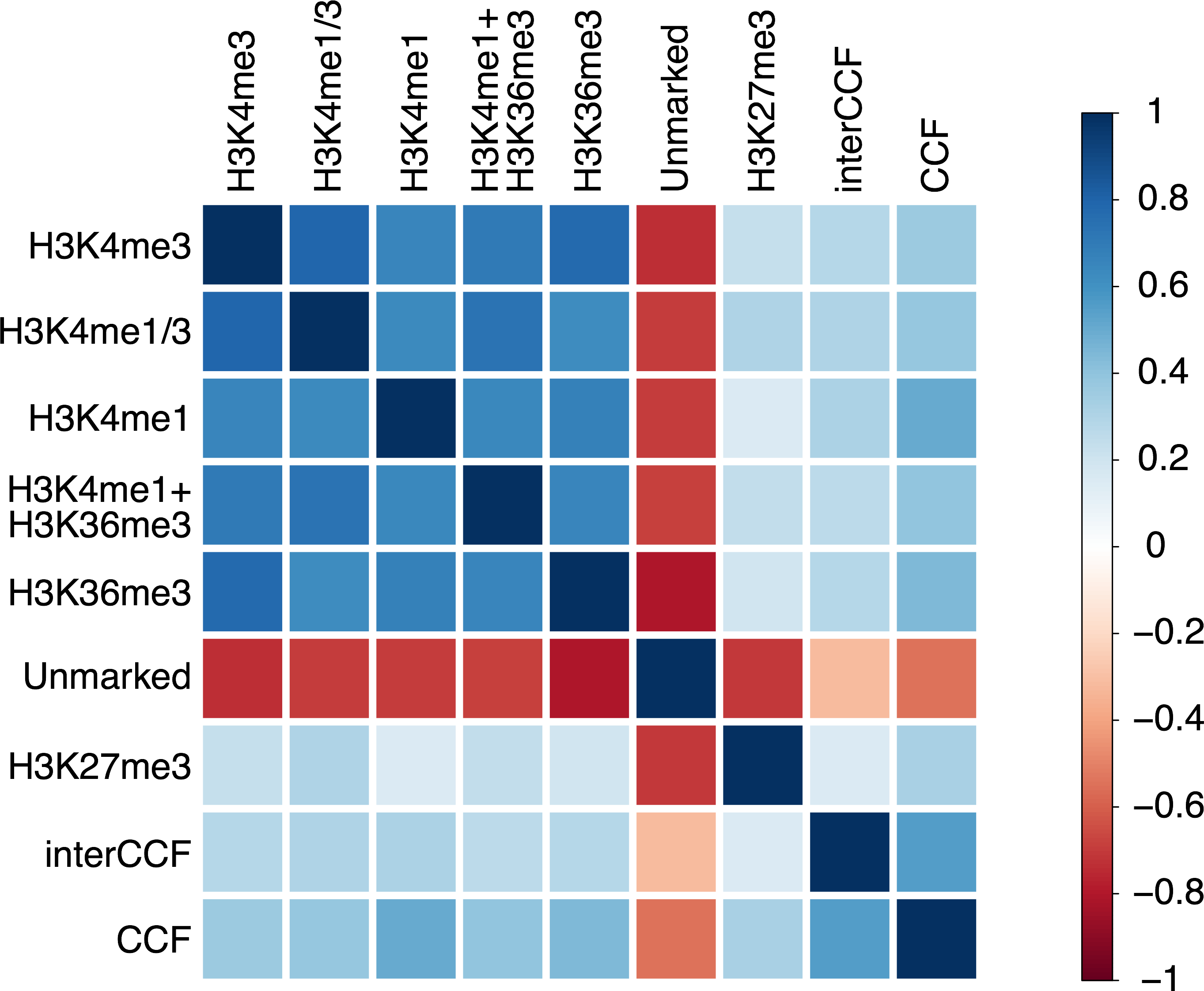


**Supplemental Figure S7. Correlation patterns of whole-genome CCF, interCCF, and chromatin states in mouse.** Mouse cell line CH12-LX. Resolution of the Hi-C map is 1 Mb.


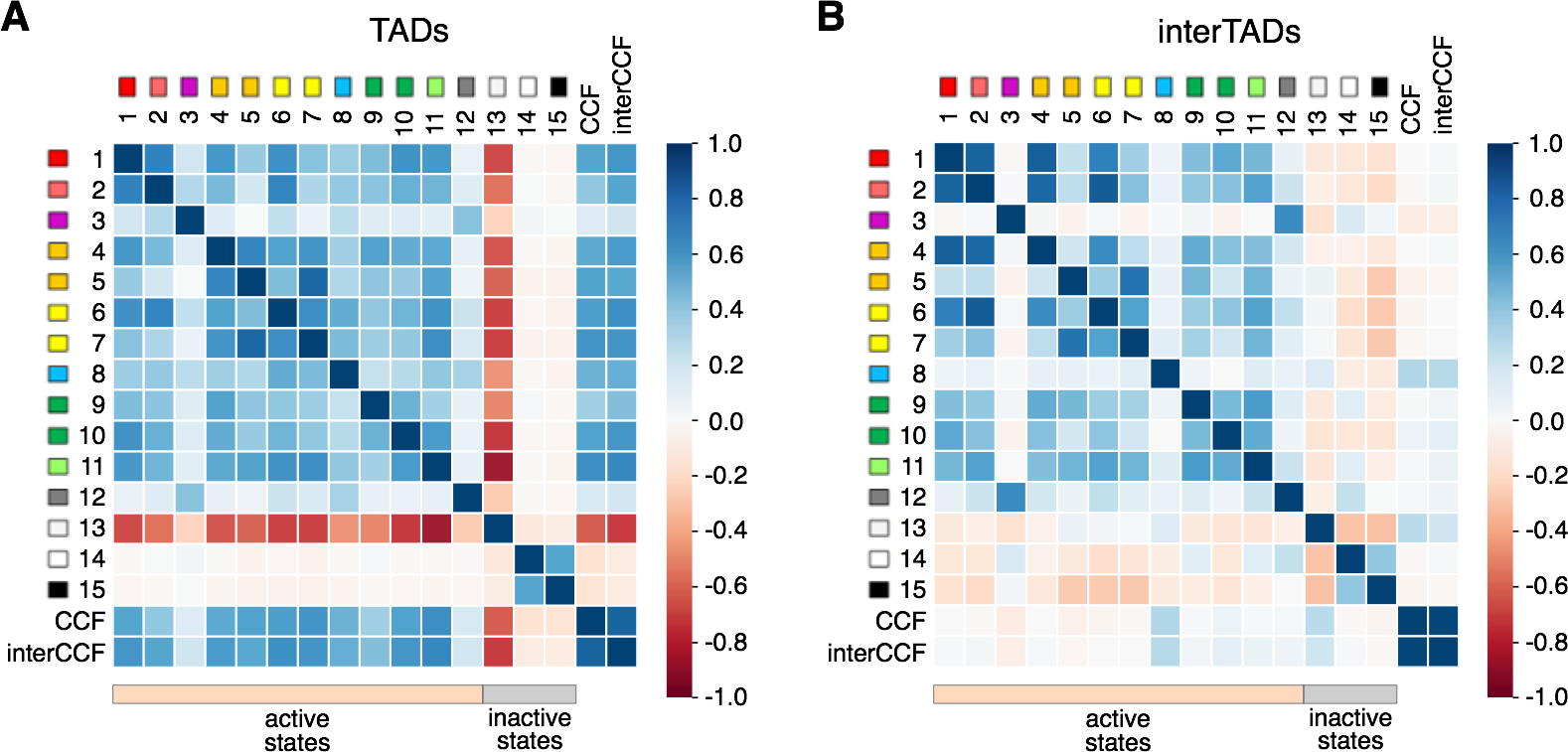


**Supplemental Figure S8. Correlation patterns for TAD and interTAD regions in human.** (A) Correlation patterns between two types of CCF and chromatin states for the whole-genome TADs (found using the *Armatus* algorithm, *gamma* = 1.0). (B) Correlation patterns for the whole-genome interTADs (all regions between TADs found using the *Armatus* algorithm, *gamma* = 1.0). Human cell line HMEC.


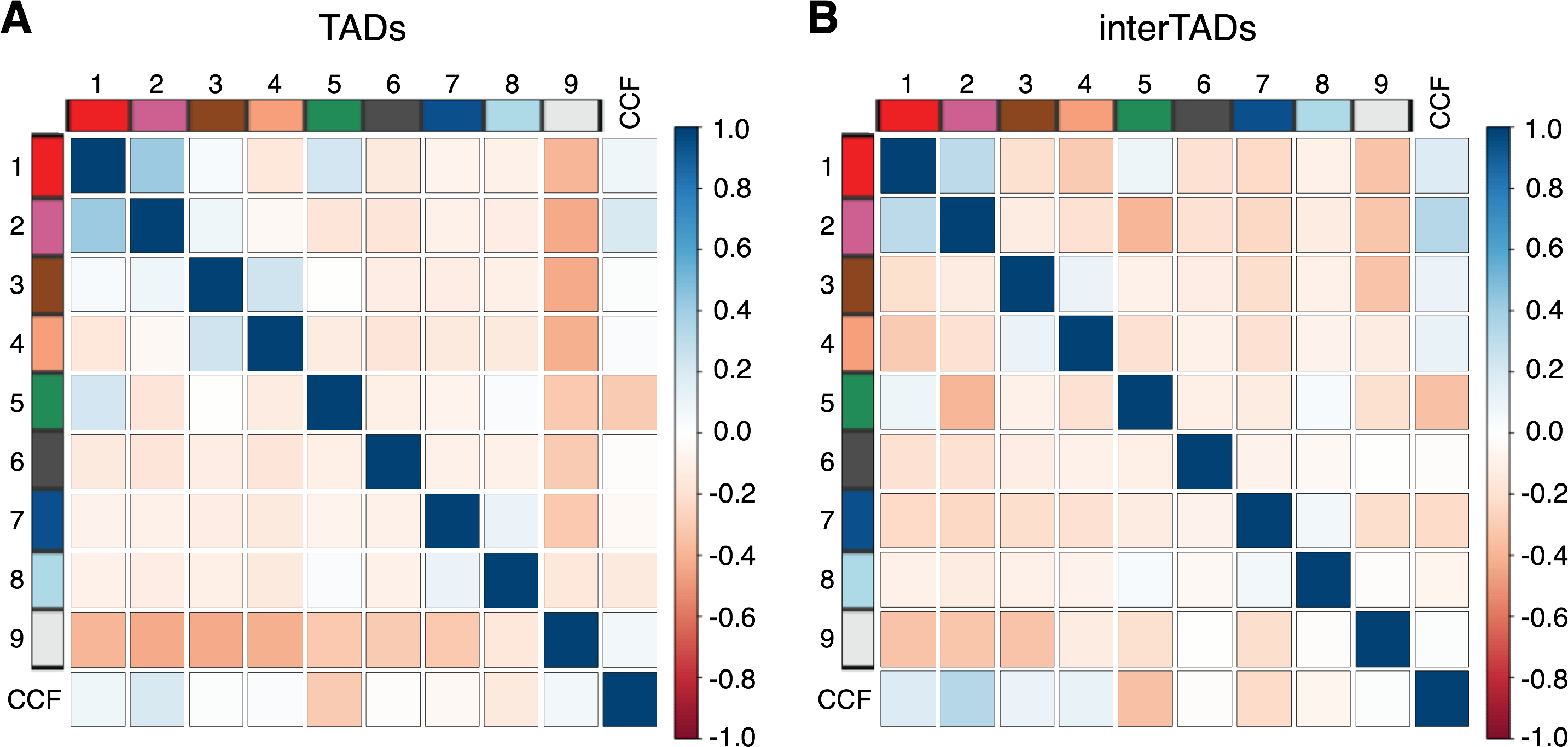


**Supplemental Figure S9. Correlation patterns for TAD and interTAD regions in *Drosophila*.** (A) Correlation patterns between whole-genome CCF and chromatin states (states 1 to 9) for the whole-genome TADs (found using the Armatus algorithm, *gamma* = 1.0). (B) Correlation patterns for the whole-genome interTADs (all regions between TADs found using the Armatus algorithm, *gamma* = 1.0). *Drosophila* cell line S2-DGRC.


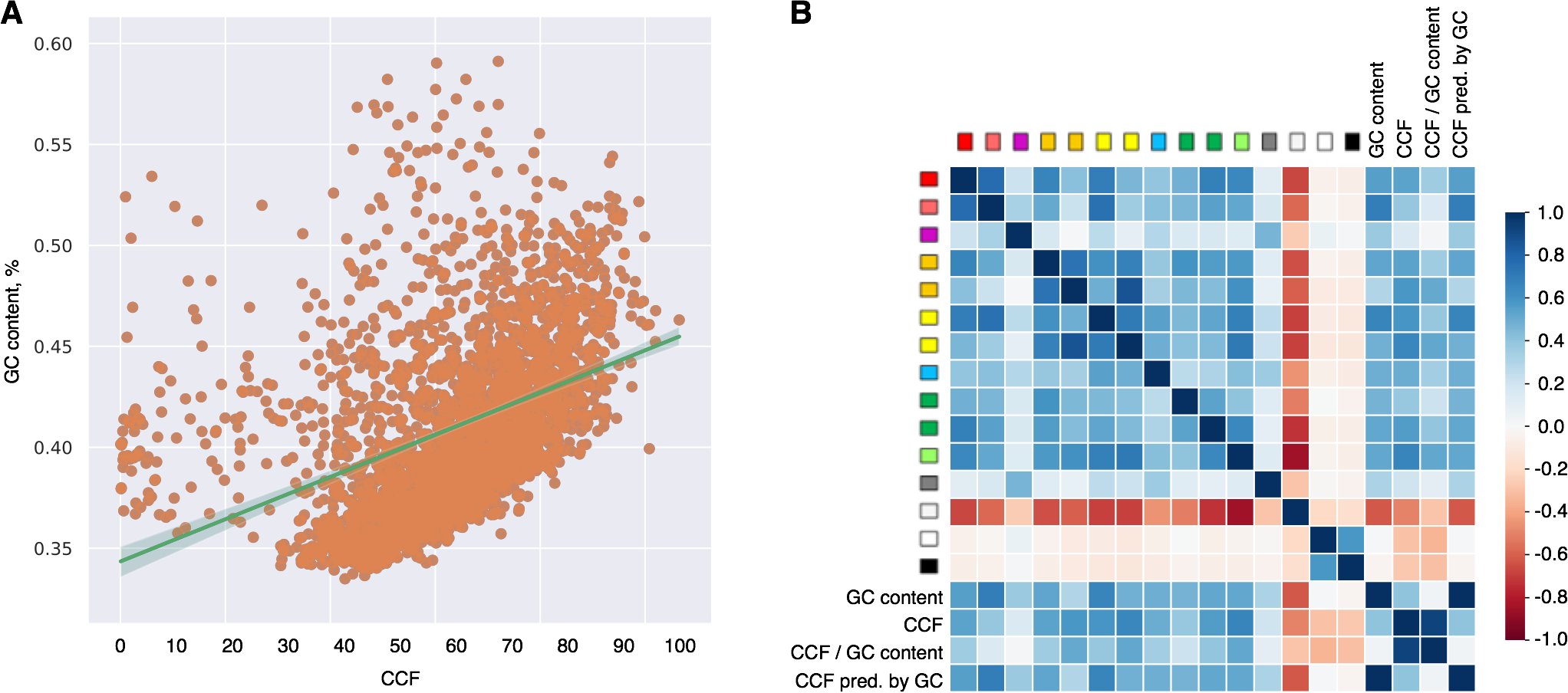


**Supplemental Figure S10. Whole-genome correlation patterns are not driven by GC-content.** (A) GC-content and whole-genome CCF are highly correlated (Pearson’s R=0.41). Orange circles show GC-content values in each genomic 1 Mb bin. The green line represents the linear regression, which was further used to predict CCF in B. The area around the line represents the confidence interval. (B) The Pearson correlation coefficients between the 15 chromatin states, GC-content, CCF, CCF divided by the GC-content, and CCF predicted by the GC-content using the linear regression shown in A.


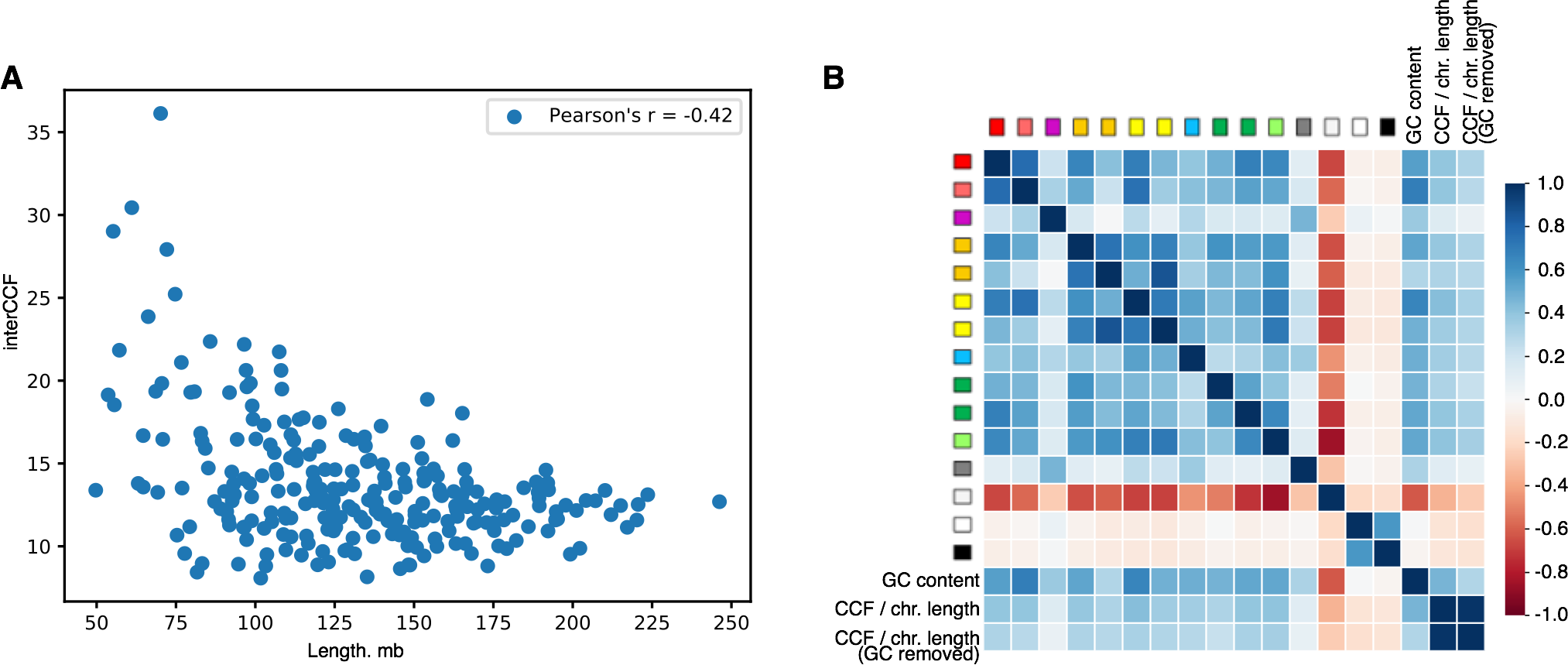


**Supplemental Figure S11. Whole-genome correlation patterns are not driven by chromosome length.** (A) Correlation of inter-chromosomal CCF with the chromosome length (an average length of two interacting chromosomes). The Pearson correlation coefficient is specified on the plot. (B) The Pearson correlation coefficients between the 15 chromatin states, GC-content, CCF normalized by the chromosome length, and CCF normalized by the chromosome length with subsequent removal of the GC-content dependency by additional normalization for GC-content. Human cell line HMEC.

**
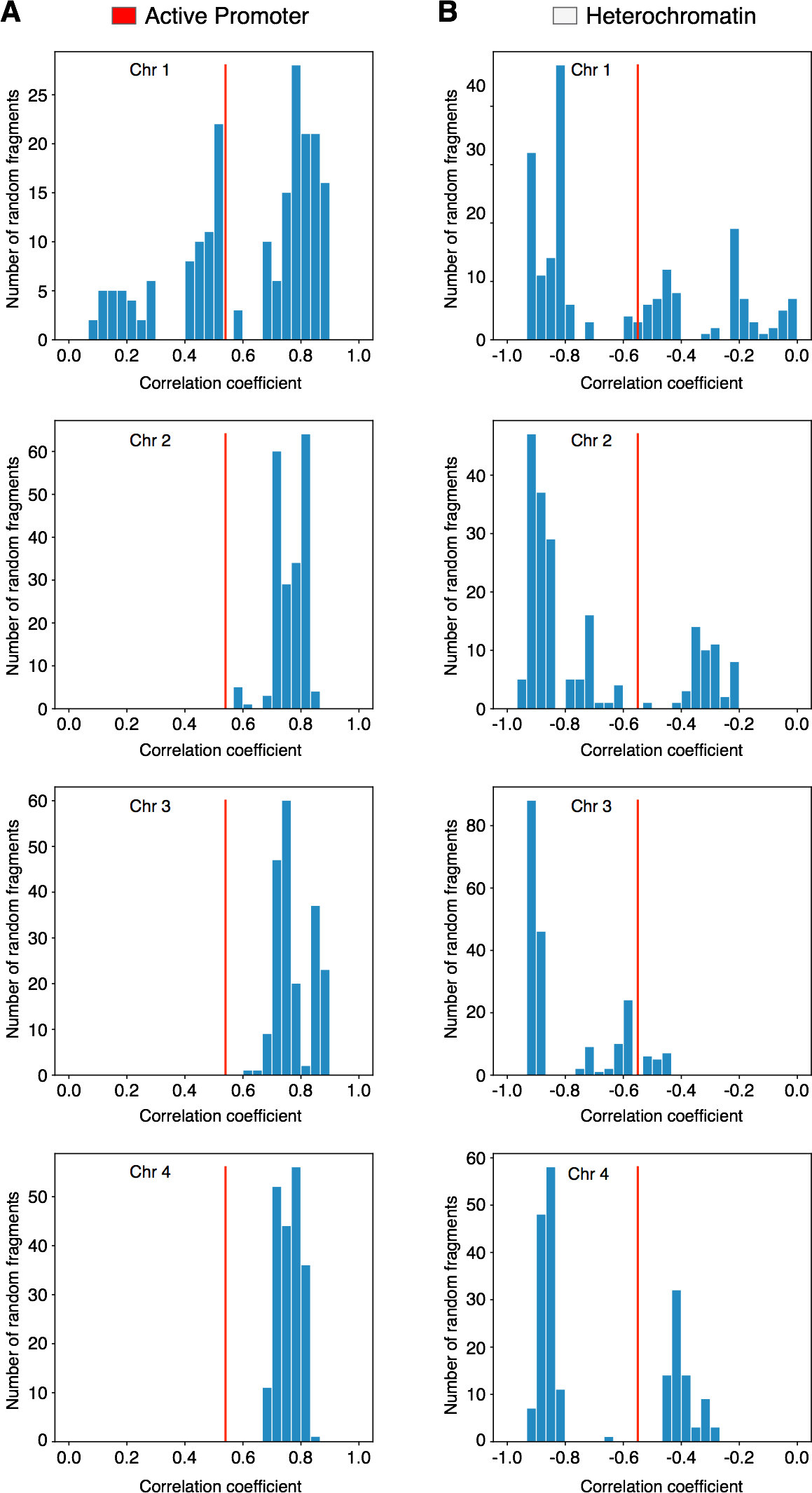
**

**Supplemental Figure S12. Correlation for the human chromosome 22 is compared with correlations for random fragments of a large chromosome equal in length (50 Mb).** (A) Distribution of correlations between inter-chromosomal CCF and active promoter chromatin state for random fragments of chromosomes 1-4 (blue histograms) compared with the real correlation for the chromosome 22 (red line). (B) Distribution of correlations between inter-chromosomal CCF and heterochromatin state for random fragments of chromosomes 1-4 (blue histograms) compared with the real correlation for the chromosome 22 (red line). Human cell line HMEC.


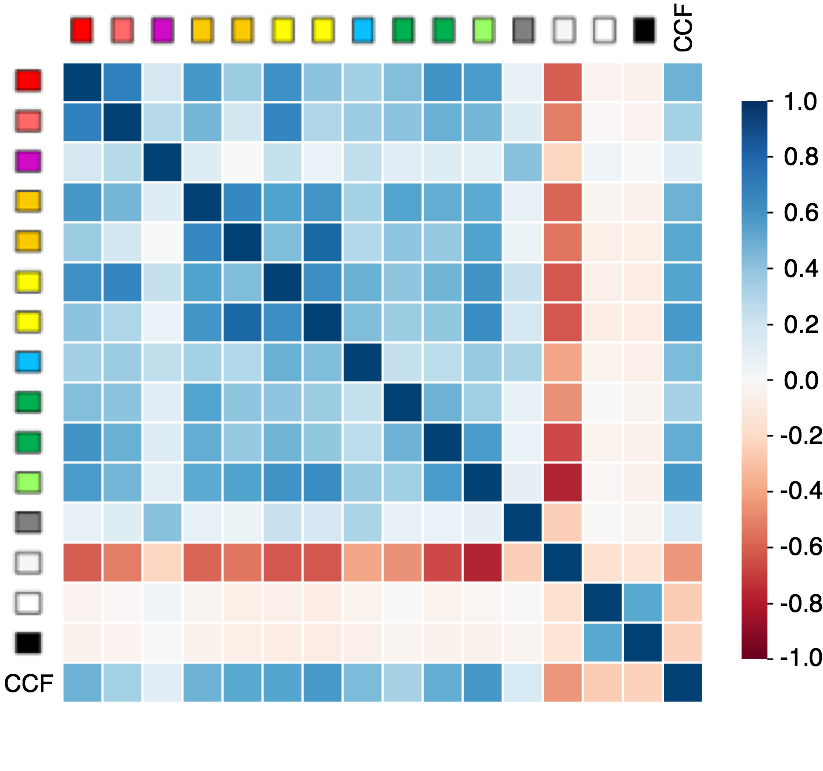


**Supplemental Figure S13. Correlation patterns for the whole human genome with centromeres excluded.** Correlation patterns between total CCF and chromatin states 1 to 15 for the whole genome with centromeres excluded at each chromosome. Human cell line HMEC.

**
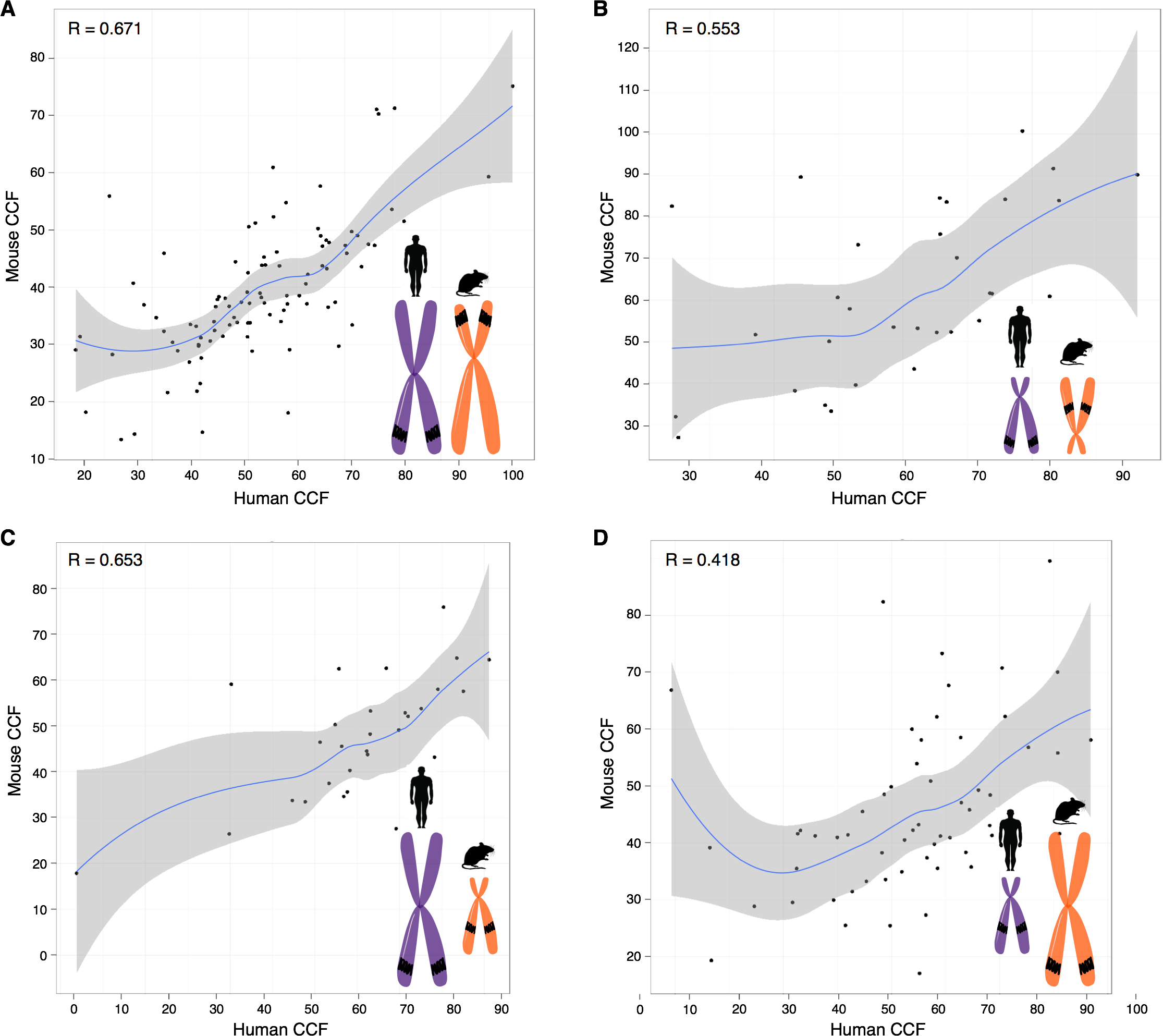
**

**Supplemental Figure S14. Contact frequency for chromatin regions is conserved in syntenic transitions between the mouse and human genomes.** (A) CCF in human versus CCF in mouse for syntenic regions in large human chromosomes and large mouse chromosomes. (B) CCF in human versus CCF in mouse for syntenic regions in small human chromosomes and small mouse chromosomes. (C) CCF in human versus CCF in mouse for syntenic regions in large human chromosomes and small mouse chromosomes. (D) CCF in human versus CCF in mouse for syntenic regions in small human chromosomes and large mouse chromosomes. Each dot represents a syntenic region of size 2 Mb and larger obtained from the Mouse Genome Informatics database (MGI). Cell lines HMEC (human) and CH12-LX (mouse). Pearson’s R is specified on each plot.

A

B


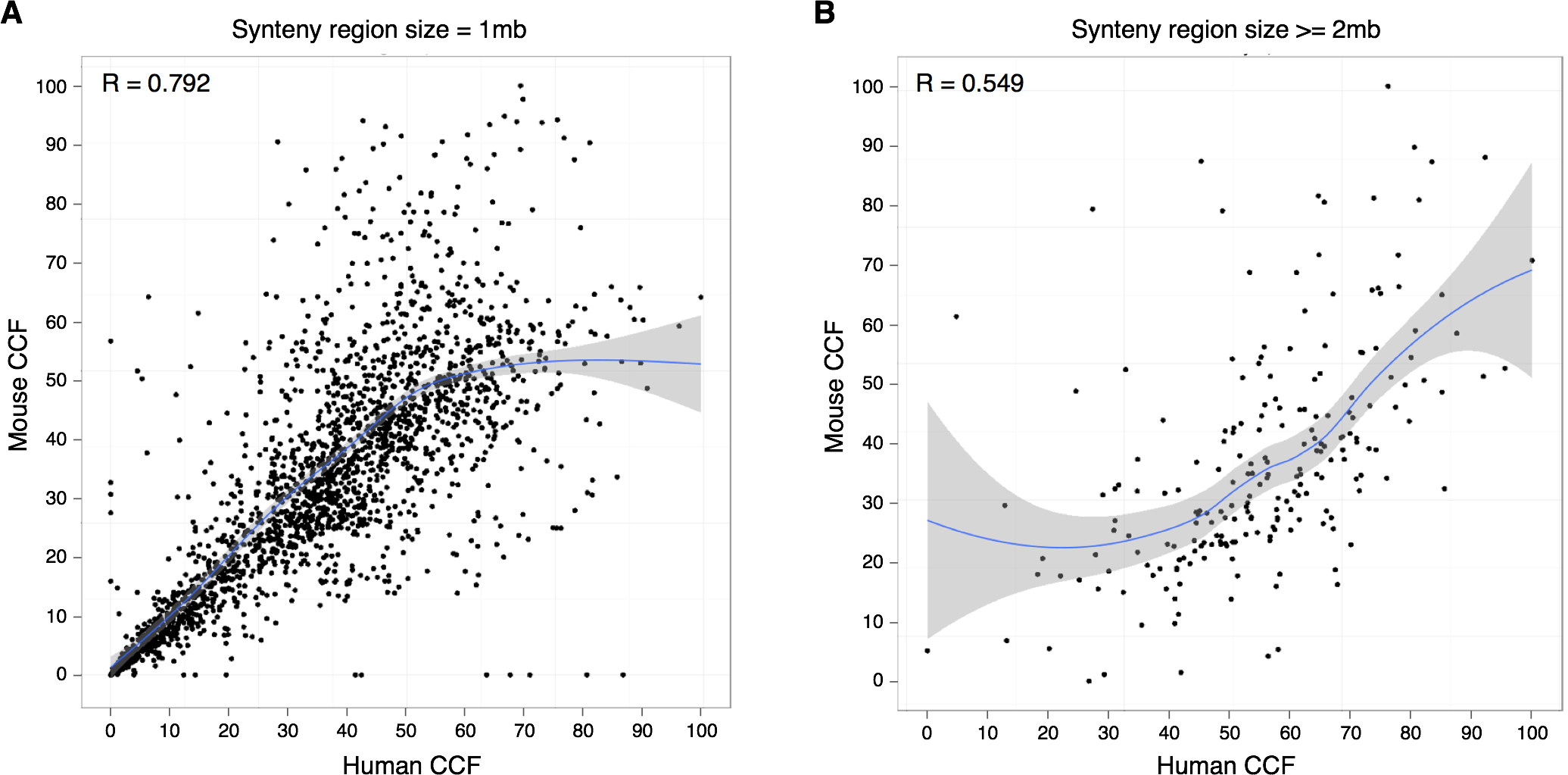


**Supplemental Figure S15. Contact frequency for syntenic regions in the mouse and human genomes.** CCF in human versus CCF in mouse is shown. (A) All syntenic regions are obtained by mapping the mouse genome to the human genome using the Liftover tool. Each dot represents a syntenic region (size 1 Mb). (B) All syntenic regions of size 2 Mb and larger obtained from the Mouse Genome Informatics database (MGI) are displayed. Each dot represents a syntenic region. Cell lines HMEC (human) and CH12-LX (mouse).
